# The Healing Process and Functional Recovery of Neuroretina after Idiopathic Macular Hole Surgery without Internal Limiting Membrane Reversal Tamponade

**DOI:** 10.1155/2020/2478943

**Published:** 2020-05-04

**Authors:** Xinlei Zhu, Jiaxing Wang, Jianan Li, Haoyu Chen, Bo Huang, Hua Yan

**Affiliations:** ^1^Department of Ophthalmology, Tianjin Medical University General Hospital, Tianjin 300052, China; ^2^Laboratory of Molecular Ophthalmology, Tianjin Medical University, Tianjin 300070, China; ^3^Department of Ophthalmology, Emory University, Atlanta, GA 30322, USA; ^4^Joint Shantou International Eye Center, Shantou University & the Chinese University of Hong Kong, Shantou 515063, China; ^5^Department of Ophthalmology, University of Mississippi Medical Center, Jackson, MS 39216, USA

## Abstract

**Purpose:**

To investigate the healing process and functional recovery of neuroretina after idiopathic macular hole surgery, as well as analyzing the influencing factors.

**Methods:**

Thirty-six eyes of 31 patients with full-thickness idiopathic macular hole (IMH) were enrolled in this retrospective study. All of them were operated using 23-gauge or 25-gauge vitrectomy with inner limiting membrane peeling and air tamponade. Spectral-domain optical coherence tomography was performed before surgery and after surgery to observe the structural changes of neuroretina.

**Results:**

Twenty eyes (55.56%) had the macular hole closed at 3 to 5 days after surgery (closed group), beginning from the inner retina based on OCT. Holes of 16 eyes (44.44%) remained unclosed and progressed to larger holes at 13 to 15 days (*t* = −2.811, *P*=0.013) after surgery (unclosed group). Compared with the eyes in the closed group, the eyes in the unclosed group had significantly larger hole diameter (*t* = −2.882, *P*=0.007). Postoperative BCVA was significantly improved in the closed group (*t* = 2.573, *P*=0.019) and not improved in the unclosed group (*t* = 0.606, *P*=0.554) at the 6-month follow-up.

**Conclusion:**

Full-thickness IMHs could achieve anatomic closure 3 to 5 days after surgery with first-step inner retina tissue bridging. Otherwise, they were not able to achieve hole closure and opened to larger holes about 2 weeks postoperatively. Macular hole diameter was an important factor affecting the healing of the holes. The delayed restoration of fovea detachment and ellipsoid area deficiency were responsible for poor vision outcomes after surgery.

## 1. Introduction

Idiopathic macular hole (IMH) is an eye disease which severely threatens the patient's vision and quality of life. It has been reported that the prevalence of idiopathic macular hole is about 0.16%–0.4% in different studies [1–3]. Several studies have described the process of spontaneous closure of IMHs assessing by OCT [4–7]. However, only 4%–6% of full-thickness MHs can resolve spontaneously without any treatments [8, 9].

Surgical treatment of idiopathic macula hole by vitrectomy was first operated by Kelly and Wendel in 1991 [10]. The surgical method of internal limiting membrane (ILM) peeling was described by Eckardt et al. in 1997 which improved the closure rate of idiopathic macular holes [11]. In 2010, Michalewska et al. proposed inverted internal limiting membrane flap technique for the treatment of large macular holes [12], which became a general practice by most surgeons in recent years. However, the necessity of performing inverted ILM flap technique was debatable. Yamashita et al. had reported that inverted ILM flap technique could improve the success rate of surgery, but the differences were not statistically significant [13, 14]. Currently, vitrectomy combined with internal limiting membrane peeling and intraocular tamponade was an effective way to close the hole and improve the visual acuity. The current closure rate of IMHs was 85%–100% after one operation [15–17]. The healing process and functional recovery of neuroretina after surgery were affected by a variety of factors. Chhablani et al. had shown that minimum diameter between the edges and longest diameter of the hole might be the best predictors of hole closure [18]. Tognetto et al. pointed out that the healing process also depended on the stage of macular holes, duration of symptoms, and surgery technique [19]. Later on, Liu et al. introduced a concept of macular hole closure index as an OCT factor related with anatomical outcome after IMH surgery [20]. Faria et al. found that anatomical and visual results were associated with how ILM was placed over the hole [21]. Besides, other studies had shown that the restoration of the external limiting membrane and the foveal cone outer segment tips (COST) line defect was related to functional recovery after IMH surgery [22, 23]. However, the healing process of IMHs after surgery and reasons why part of IMHs remained unclosed had not been described clearly yet.

In the current study, we would like to demonstrate both structural and functional healing of full-thickness IMHs after surgery, as well as analyzing the factors which caused full-thickness IMHs remained unclosed in this population.

## 2. Patients and Methods

The study was approved by the Tianjin Medical University General Hospital medical ethics committee. All participants had signed informed consent according to the Declaration of Helsinki. We enrolled 36 eyes (31 patients) with full-thickness IMH undergoing surgical treatment in Tianjin Medical University General Hospital (Tianjin, China) from August 2015 to November 2018. Patients with history of ocular trauma, high myopia, optical media opacity, glaucoma or other optic nerve disease, and other retinopathy were excluded. Besides, patients with macular hole caused by high myopia or ocular trauma were also not included.

All the surgeries were performed by a single experienced ophthalmologist. The 23-gauge or 25-gauge trocars were used for surgical incision. The surgical procedures included three-port pars plana vitrectomy (PPV), posterior vitreous detachment, ILM staining with indocyanine green, subsequent ILM peeling, fluid-air exchange, and room air-filling, except ILM reversal tamponade. The range of ILM peeling was approximately two to three disc diameters (DD). However, cataract surgery was not combined with pars plana vitrectomy at the same time. All patients were instructed to keep face-down position for 3 days after surgery and avoided the supine position until resolution of the intraocular air. Eyes were divided into two groups. Closed group were eyes which achieved anatomic closure of the holes after surgery based on OCT, while the eyes with holes remained unclosed 6 months after surgery were in the unclosed group.

Complete ophthalmic examinations were performed, which included best corrected visual acuity (BCVA), intraocular pressure (IOP), slit lamp inspection, direct/indirect ophthalmoscope, and OCT before and after surgery. BCVA was obtained by the standard logarithmic visual acuity chart and represented by a logarithm of the minimal angle of resolution (LogMAR) for statistical analysis. OCT examination was acquired for all the visits using CIRRUS SD-OCT 4000 (Carl Zeiss, Meditec, Germany) with a central wavelength of 840 nm by the same technician. IMHs were staged according to Gass's classification and International Vitreomacular Traction Study (IVTS) Group classification [14, 24]. Macular hole diameter (the smallest hole diameter), hole basal diameter (the distance of edge at the base of the hole), the length of the ellipsoid area defect, and hole height were measured on the OCT B-scan across fovea before surgery and compared between two groups. The first OCT and BCVA were obtained 3–5 days after surgery when the air bubble was absorbed, and macula was visible by direct/indirect ophthalmoscope. So, the first data after surgery was different for each patient as the air absorption rate varies from patient to patient. All the patients were followed for 6 months with two-week interval with OCT and BCVA acquired.

SPSS 20.0 was used for statistical analysis. The measurement results (age, AV, and OCT measurements) were presented as mean ± standard deviation (SD). The preoperative and postoperative BCVA was analyzed by the paired *t* test. The diameter of macular hole in the unclosed group before surgery and 2 weeks after surgery were also analyzed by the paired *t* test. The macular hole diameter, hole basal diameter, the lengths of the ellipsoid area defect, and hole height of the closed group and unclosed group were compared by the two-sample *t*-test. Using Fisher's exact test, the relationship between the stage and healing state of full-thickness IMH was determined. *P* ≤ 0.05 was considered statistically significant.

## 3. Results

### 3.1. Clinical Characteristic

Thirty-one full-thickness IMH patients were enrolled in our study. The mean age of patients was 66.08 years old with a range of 54 to 81 years old. Nine (29.03%) patients were male and 22 (70.97%) patients were female. Twenty of 36 eyes (55.56%) achieved anatomic closure of the holes after surgery based on OCT (Table 1). While, holes of the rest 16 (44.44%) eyes remained unclosed at the latest follow-up which was 6 months after surgery (Table 2). Only one patient with unclosed macular hole accepted second operation with the same surgical procedures as first operation. However, the macular hole remained unclosed yet. No complications that required medical or surgical intervention were observed.

### 3.2. Anatomy Features of Healing Process

**Figure 1:**
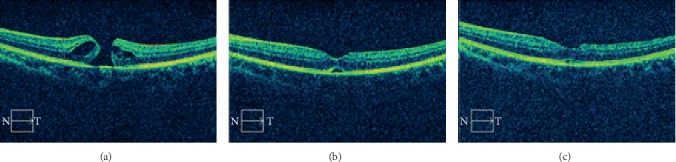
The OCT B-scan across the fovea of a 54-year-old male's left eye with full-thickness idiopathic macular hole. (a) OCT B-scan at 1 week before surgery showing full-thickness macular hole (diameter = 341 *μ*m) and intraretinal cysts (BCVA = 0.52). (b) OCT B-scan at 3 days after surgery showing the disappearance of intraretinal cysts and the inner retinal tissue connection like a bridge with a subfoveal space (BCVA = 0.22). (c) OCT B-scan at 2 months after surgery showing the resolve of subfoveal space and well-arranged ellipsoid area (BCVA = 0.10).

**Figure 2:**
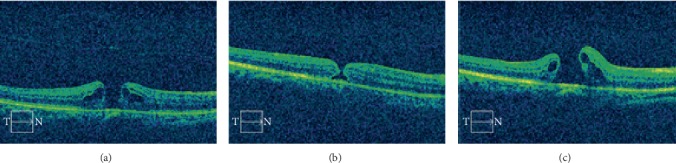
The OCT B-scan across the fovea of a 65-year-old female's right eye with full-thickness idiopathic macular hole. (a) OCT B-scan at 2 days before surgery showing full-thickness macular hole (diameter = 414 *μ*m) and intraretinal cysts (BCVA = 1.10). (b) OCT B-scan at 5 days after surgery showing the disappearance of intraretinal cysts and a smaller distance between the edges of the hole (BCVA = 1.10). (c) OCT B-scan at 13 days after surgery showing the reappearance of intraretinal cysts and further opening of the hole (BCVA = 1.00).

The healing of IMH after surgery began from the inner retina with cystoid changes resolved and the hole diameter reduced in both groups 3–5 days after surgery. In all the eyes in the closed group, inner retina tissue bridging seemed like a crucial early step for hole closure, which could be observed at 3–5 days after surgery (Figure 1, Table 1). Conformingly, all the eyes without tissue bridge forming 1 week after surgery were not able to achieve hole closure at the follow-up visits. Instead, the intraretinal cysts reappeared and led to further opening of the hole to a larger one (*t* = −2.811, *P*=0.013) (Figure 2), which usually happened at 13–15 days after surgery (Table 2). The outer fovea defect/detachment could be persistent for 1–12 weeks and could be finally resolved with or without ellipsoid area deficiency (Figure 1).

Compared with the eyes in the closed group, the eyes in the unclosed group had significantly larger hole diameter (*t* = −2.882, *P*=0.007), shown in Table 3. Although the value of hole basal diameter, the lengths of the ellipsoid area defect, and hole height were larger in the eyes of the unclosed group compared with that in the closed group, the difference was not statistically significant (*t* = −1.687, *P*=0.101; *t* = 0.654, *P*=0.517; *t* = −1.012, *P*=0.319).

According to Gass's classification, idiopathic macular holes could be divided into four stages. In the closed group, 50.00% and 45.00% eyes had macular holes in stage II and III, respectively. Only 5.00% eyes had macular holes progressed to stage IV before surgery. However, this proportion accounted for 56.25% in the unclosed group. The differences were statistically significant (*χ*^2^ = 11.635, *P*=0.004). In the classification proposed by the IVTS group, there were no significant differences between the closed group and the unclosed group (*χ*^2^ = 5.096, *P*=0.066) (Table 4).

### 3.3. Vision Prognosis

There was no significant difference of preoperative BCVA between two groups (*t* = −1.979, *P*=0.058). BCVA of the eyes in the closed group was significant improved (*t* = 2.573, *P*=0.019). Of the 20 eyes, the BCVA improved in 16 (80.00%) eyes, remained unchanged in 2 (10.00%) eyes, and decreased in 2 (10.00%) eyes after surgery (Table 1). However, no significant change in BCVA was found in the eyes of the unclosed group after surgery (*t* = 0.606, *P*=0.554) (Table 2).

## 4. Discussion

Vitrectomy and ILM peeling is currently considered an effective treatment for IMH [25]. It had shown that it can significantly improve the success rate of macular hole surgery [26]. In our study, we included only 36 eyes of 31 patients with full-thickness IMH, which was one of the limitations of our study. All of them underwent PPV combined with ILM peeling, fluid-air exchange, face-down position for 3 days after surgery, and avoided the supine position until resolution of the intraocular air. Even several groups had reported that vitrectomy without face-down position could also receive satisfactory anatomical recovery and significant improvement of visual acuity [27–29]. Twenty patients (55.56%) had successful anatomical closure of the hole after surgery, and 16 patients (80.00%) had improvement of VA which was similar to the results in previous reports [15–17].

Based on the data we have, we found that macular holes could achieve anatomic closure in 3–5 days after surgery. Otherwise, they were not able to achieve hole closure and furthermore opened to larger holes about 2 weeks postoperatively. We believed that it was worthy to pay close attention to the changes in the macular hole within one week after surgery, and if they were not able to close in 1 week after surgery, the chance of healing for holes was little. One week after surgery was a time window for some future positive treatments intervened for the holes with no tendency of closure.

We began to observe the structural recovery process of full-thickness IMH using OCT 3 to 5 days after surgery, which was one of the limitations of the current study. We found that the neuroretina layer around the macular hole grew into the center 3 to 5 days after surgery and formed a connection with each other which was like a bridge connecting two edges. It may happen as early as 24 hours postoperatively, according to several other reports [20, 30]. At this moment, the neuroretina had not adhered to retina pigment epithelium (RPE) and subretinal fluid (SRF) could be found on OCT B-scan. The SRF could be resorbed gradually with time leading to the complete hole closure. Researchers had shown that müller cells and glial cells were important factors in promoting hole healing histologically [4, 31, 32]. According to our research and previous literatures [30, 33], it was possible to divide full-thickness IMH healing into the following 3 stages. Stage I: with the traction from ILM to the neuroretina released after surgery, intraretinal cysts got resolved. Stage II: the inner retinal layers grew to the center of the macular hole and two edges were connected by forming a tissue bridge. At this stage, SRF could still be found on B-scan. Stage III: the SRF was resolved and photoreceptors begin remodeling, which could lead to a complete ellipsoid area.

In our study, the IMHs of 16 (44.44%) eyes remained unclosed postoperatively, which received furthermore ILM peeling or ILM flap insert or covering technique lately. These unclosed IMHs had significantly larger hole diameter (*t* = −2.882, *P*=0.007) compared with those in the closed group. In addition, eyes in the unclosed group had a higher proportion of macular holes in stage IV (*χ*^2^ = 11.635, *P*=0.004). Therefore, we did not recommend only using PPV and ILM peeling to treat the stage IV macular hole. Combined inverted ILM flap technique could be a good choice. It was possible to consider that the macular hole diameter was a crucial factor for IMHs closure. The unclosed IMHs had a tendency to close in the early days after surgery. The inner retinal layers moved towards the center of the hole gradually with a reduction in the diameter of the hole. However, without retina tissue bridging, this early close tendency was followed by further opening of the hole and perifoveal pseudocysts forming as shown in Figure 2. Yuksel et al. reported that perifoveal pseudocysts were related to the macular hole unclosed, which was consistent with our results [34]. Smiddy and Flynn found that early macular hole could be self-repaired through retinal tissue junction [35]. But if the repair failed, retinal glial cells would move to the edge of the hole and cause the hole increase gradually by shrinking. They furthermore demonstrated that retina tissue connection and macular hole diameter were the important factors affecting hole healing. There was one more possible reason for the unclosed hole. The room air in the vitreous cavity was absorbed faster in some eyes causing less oppressive effect on the hole. The reason was due to individual variance, which was difficult to control or measure.

In our study, BCVA of the eyes in the closed group was significant improved (*t* = 2.573, *P*=0.019), but no significant change in BCVA was found in eyes of the unclosed group after surgery. The delayed restoration of fovea detachment and ellipsoid area deficiency were responsible for poor vision outcomes after surgery. This was consistent with some other papers [22, 36]. It had been reported that the recovery of the external limiting membrane (ELM) and foveal cone outer segment tips (COST) line defect were related to the visual recovery after IMH surgery [23, 37].

## 5. Conclusions

In conclusion, full-thickness IMHs could achieve anatomic closure 3–5 days after surgery with first-step inner retina tissue bridging. Otherwise, they were not able to achieve hole closure and opened to larger holes about 2 weeks postoperatively. Preoperative macular hole diameter was an important factor affecting the healing of the holes. It was not recommended only using PPV and ILM peeling to treat the stage IV macular hole. The delayed restoration of fovea detachment and ellipsoid area deficiency were responsible for poor vision outcomes after surgery.

## Figures and Tables

**Table 1:** Characteristic of patients in the closed group.

Case no.	Gender	Age (years)	Systemic disease	Macular hole diameter (*μ*m)	Preoperative BCVA (LogMAR)	Postoperative BCVA (LogMAR)	Time of first OCT after surgery (days)
1	Male	54	No	341	0.52	0.10	3
2	Male	70	Yes	399	0.92	0.52	4
3	Female	57	Yes	464	0.70	0.70	3
4	Female	63	Yes	548	0.92	0.70	3
5	Male	66	Yes	444	0.82	0.70	5
6	Female	66	No	325	0.70	0.40	3
7	Female	70	No	504	0.82	1.00	3
8	Male	70	No	341	0.70	0.70	4
9	Female	66	Yes	473	1.10	0.60	4
10	Female	67	Yes	208	0.70	0.52	4
11	Male	57	No	400	1.00	0.92	3
12	Female	56	Yes	785	2.00	1.22	3
13	Male	80	No	474	1.22	1.00	3
14	Female	65	No	341	1.10	2.00	3
15	Female	64	No	252	0.60	0.52	5
16	Female	67	No	311	1.10	0.52	4
17	Female	62	Yes	310	0.92	0.82	4
18	Female	62	Yes	710	1.70	0.92	4
19	Female	62	Yes	448	1.40	1.00	3
20	Male	72	Yes	134	0.82	0.70	3
Mean ± SD	—	64.80 ± 6.00	—	410.60 ± 150.92	0.99 ± 0.36	0.78 ± 0.37	3.55 ± 0.67

All eyes in the closed group were phakic.

**Table 2:** Characteristic of patients in the unclosed group.

Case no.	Gender	Age (years)	Systemic disease	Macular hole diameter (*μ*m) before surgery	Preoperative BCVA (LogMAR)	Postoperative BCVA (LogMAR)	Macular hole diameter (*μ*m) 13–15 days after surgery
1	Female	68	Yes	458	0.92	1.00	623
2	Female	65	No	414	1.10	1.00	650
3	Female	57	Yes	626	1.00	0.7	909
4	Female	69	Yes	682	0.82	0.92	897
5	Female	69	No	554	1.00	0.82	356
6	Male	81	Yes	630	2.00	2.00	768
7	Male	72	Yes	639	0.82	2.00	591
8	Male	72	Yes	429	0.52	0.3	770
9	Female	67	No	931	0.92	0.70	1081
10	Female	64	Yes	591	1.70	0.82	813
11	Male	58	Yes	311	0.92	0.70	621
12	Female	76	Yes	222	1.40	2.00	488
13	Female	62	No	1321	2.00	2.00	1280
14	Female	61	Yes	815	1.70	1.22	990
15	Female	63	No	384	1.70	1.22	739
16	Female	79	Yes	903	2.00	2.00	562
Mean ± SD	—	67.69 ± 6.82	—	619.38 ± 265.81	1.28 ± 0.48	1.21 ± 0.57	758.63 ± 227.19

No. 8 eye was pseudophakic, and others were phakic.

**Table 3:** Comparison of the closed group and unclosed group.

Comparison factors	Closed group (mean ± SD)	Unclosed group (mean ± SD)	*P*
Age	64.80 ± 6.00	67.69 ± 6.82	0.187^*∗*^
Gender	35.00% males	25.00% males	0.517^#^
Systemic diseases	55.00%	68.75%	0.400^#^
Hole diameter (*μ*m)	410.60 ± 150.92	619.38 ± 265.81	0.007^*∗*^
Hole basal diameter (*μ*m)	774.05 ± 265.63	1011.19 ± 534.07	0.101^*∗*^
Ellipsoid area defect (*μ*m)	1640.15 ± 463.76	1524.25 ± 568.77	0.517^*∗*^
Hole height (*μ*m)	409.20 ± 81.51	482.13 ± 299.78	0.319^*∗*^

^*∗*^ *t*-test. ^#^Chi-square test.

**Table 4:** The relationship between the stage and healing state of full-thickness IMH.

Classification	Macular hole closed after surgery		*P*
	Yes (*n* = 20)	No (*n* = 16)	
Gass's classification, *n* (%)			0.004
Stage I	0 (0)	0 (0)	
Stage II	10 (50.00)	3 (18.75)	
Stage III	9 (45.00)	4 (25.00)	
Stage IV	1 (5.00)	9 (56.25)	

IVTS group classification, *n* (%)			0.066
Small FTMH	2 (10.00)	1 (6.25)	
Medium FTMH	8 (40.00)	2 (12.50)	
Large FTMH	10 (50.00)	13 (81.25)	

IVTS: International Vitreomacular Traction Study; FTMH: full-thickness macular hole.

## Data Availability

The data used to support the findings of this study are available from the corresponding author upon request.
